# Clinical impact of subclonal EGFR T790M mutations in advanced-stage EGFR-mutant non-small-cell lung cancers

**DOI:** 10.1038/s41467-021-22057-8

**Published:** 2021-03-19

**Authors:** Tereza Vaclova, Ursula Grazini, Lewis Ward, Daniel O’Neill, Aleksandra Markovets, Xiangning Huang, Juliann Chmielecki, Ryan Hartmaier, Kenneth S. Thress, Paul D. Smith, J. Carl Barrett, Julian Downward, Elza C. de Bruin

**Affiliations:** 1 Translational Medicine, Oncology R&D, AstraZeneca, Cambridge, UK; 2 Bioscience, Oncology R&D, AstraZeneca, Cambridge, UK; 3 Discovery Science, BioPharmaceutical R&D, AstraZeneca, Cambridge, UK; 4 Translational Medicine, Oncology R&D, AstraZeneca, Boston, MA USA; 5 Biometrics Oncology, Oncology R&D, AstraZeneca, Cambridge, UK; 6 Oncogene Biology, Francis Crick Institute, London, UK; 7 Present Address: Global Marketing Diagnostics, Oncology Business, AstraZeneca, Gaithersburg, MD USA

**Keywords:** Translational research, Cancer genomics

## Abstract

Advanced non-small-cell lung cancer (NSCLC) patients with EGFR T790M-positive tumours benefit from osimertinib, an epidermal growth factor receptor-tyrosine kinase inhibitor (EGFR-TKI). Here we show that the size of the EGFR T790M-positive clone impacts response to osimertinib. T790M subclonality, as assessed by a retrospective NGS analysis of 289 baseline plasma ctDNA samples from T790M‐positive advanced NSCLC patients from the AURA3 phase III trial, is associated with shorter progression-free survival (PFS), both in the osimertinib and the chemotherapy-treated patients. Both baseline and longitudinal ctDNA profiling indicate that the T790M subclonal tumours are enriched for PIK3CA alterations, which we demonstrate to confer resistance to osimertinib in vitro that can be partially reversed by PI3K pathway inhibitors. Overall, our results elucidate the impact of tumour heterogeneity on response to osimertinib in advanced stage NSCLC patients and could help define appropriate combination therapies in these patients.

## Abstract (web summary)

A recent clinical trial shows that a proportion of lung cancer patients carrying the EGFR T790M mutation develop resistance to osimertinib. In this study, the authors show that T790M subclonality is associated with worse clinical outcome likely through co-occurring PIK3CA alterations, which can be targeted by PI3K pathway inhibitors.

## Introduction

Identification of genomic drivers of non-small-cell lung cancer (NSCLC) has led to the development of targeted therapies, such as tyrosine kinase inhibitors (TKIs) for activating mutations in the epidermal growth factor receptor (EGFR) gene. However, cancer cells often develop various strategies to protect themselves from such personalised therapies. This therapy-induced cancer evolution leads to an occurrence of genomically diverse subclones within a single tumour followed by a therapeutic resistance^1,2^. The EGFR T790M secondary mutation is the most common resistance alteration in EGFR-mutant NSCLC patients treated with the first- or second-generation TKIs^3–6^. The current standard-of-care for such patients is the use of the third-generation EGFR-TKI osimertinib, which irreversibly inhibits the activity of both the EGFR-activating (L858R, exon 19 deletion) and resistance (T790M) mutations while having weaker inhibitory activity against wild-type EGFR^7,8^.

Some evidence suggests that the T790M clonality level, i.e. the size of the T790M-positive population of tumour cells, might influence response to the third-generation TKIs^9–11^. Retrospective genotyping of matched tumour biopsy and plasma samples from patients enrolled in the osimertinib first-in-man study (AURA, NCT01802632) led to an identification of patients with T790M-negative tumours, but T790M-positive plasma, indicating that the mutation was only present in a fraction of tumour cells in these patients^9^. Interestingly, this subset of patients showed the shortest PFS and a lower objective response rate. In addition, patients who had lost the T790M mutation at progression had significantly shorter PFS and these patients tended to have a smaller fraction of T790M over the activating EGFR mutation in their tumours at baseline^11^.

In the AURA3 phase III trial (NCT02151981), osimertinib showed a statistically significant and clinically meaningful improvement in PFS over platinum-based doublet chemotherapy in advanced NSCLC patients whose tumours had progressed on prior EGFR-TKI therapy and were positive for T790M^12^. Despite the impressive response rates, a high proportion of patients eventually developed resistance to osimertinib, leading to disease progression. Given that T790M-positive status was a key inclusion criterion for AURA3, data from this study are ideal for studying the interplay between T790M subclonality (i.e. the presence of T790M in only a small fraction of tumour cells) and response to osimertinib.

Here, we retrospectively analyse baseline plasma from patients enrolled on the AURA3 clinical study to identify patients with subclonal T790M and show that subclonal T790M genotype is associated with shorter PFS in osimertinib-treated patients. Interestingly, these samples were enriched for co-occurring activating PIK3CA mutations, which we demonstrate to reduce sensitivity to osimertinib in vitro in EGFR-mutant cell lines. Overall, our results shed light on the implications of T790M subclonality in advanced-stage NSCLC patients and its link to resistance to osimertinib and could help define appropriate combination therapies in these patients.

## Results

### Mutant EGFR shedding into plasma at baseline

**Fig. 1:**
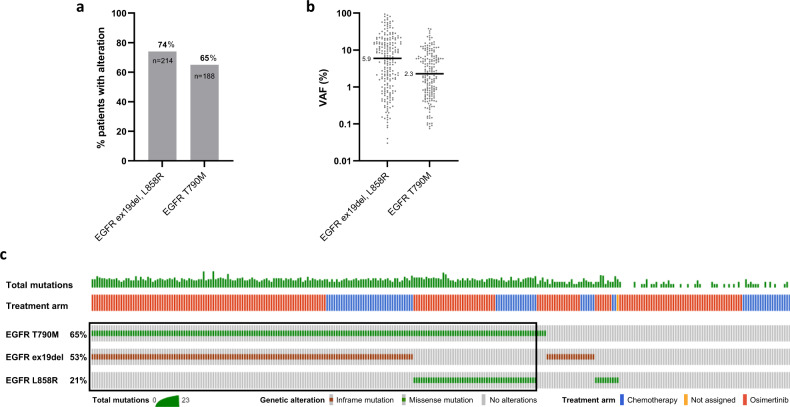
Known EGFR alterations detectable in plasma cfDNA at baseline by next-generation sequencing. **a** Percentage of patients with detectable activating EGFR mutations, either exon 19 deletion (ex19del) or L858R, and T790M. Both treatment arms, osimertinib and platinum‐based therapy plus pemetrexed, are included in the graph. **b** Distribution of VAF of EGFR ex19del or L858R and T790M. Black line represents median VAF. **c** Oncoprint showing concurrent EGFR alterations detected in 289 AURA3 patients. A total number of detectable alterations per patient is shown in a histogram at the top of the oncoprint. Patients positive for both activating EGFR mutation and T790M have been selected for further analysis (*n* = 184, black rectangle).

In order to identify AURA3 patients whose tumours harbour a subclonal T790M mutation, we first evaluated the prevalence and level of detection of the activating EGFR mutations, exon 19 deletion (ex19del) and L858R, and the TKI-resistant EGFR T790M in plasma collected at baseline using a clinically validated NGS-based cell-free DNA (cfDNA) assay (Supplementary Table 1; details in ‘Methods'). Activating EGFR mutations were detected in 214 (74%) out of 289 available baseline plasma samples with median variant allele frequency (VAF) 5.9% (Fig. 1a, b). The median VAF of ex19del and L858R was comparable, 6.5% and 4.7%, respectively (Mann–Whitney test, *P* = 0.6687; Supplementary Fig. 1). EGFR T790M mutation was identified in 188 (65%) plasma samples (Fig. 1a) with median VAF 2.3% (Fig. 1b), which was significantly lower than median ex19del and L858R VAFs (*P* < 0.0001 in both comparisons, Supplementary Fig. 1).

Approximately 64% of samples (*n* = 184) had detectable levels of both the activating EGFR mutation and T790M, 34 samples (12%) were positive for either the activating EGFR mutation or T790M and 71 samples (25%) had neither detectable levels of the activating EGFR mutation nor T790M by NGS, and those patients were considered non-shedders (Fig. 1c and Supplementary Fig. 2a). Interestingly, patients positive for both the activating and resistance EGFR mutations exhibited the highest number of aberrations detectable in the 73 tested genes (Supplementary Fig. 2b) and the highest median VAFs of such genomic alterations (Supplementary Fig. 2c). Taking into account that EGFR ctDNA shedding status has been previously found to be associated with a baseline tumour target lesion size in AURA3 patients^13^, our results indicate that AURA3 patients, who are not positive for both the activating and resistance EGFR mutations, may have smaller tumours.

### EGFR T790M subclonality at baseline is associated with worse tumour response in osimertinib-treated patients

To identify patients whose tumours harbour a subclonal T790M mutation, patients with both an activating EGFR mutation and T790M detectable in cfDNA (*n* = 184) were included in the analysis (Fig. 1c and Supplementary Fig. 3). Taking into account that the activating EGFR mutations are considered an early clonal somatic event in NSCLC development^2,14^ we assumed their presence at a clonal level within the tumour and related the T790M VAF to the activating EGFR mutation VAF in order to calculate T790M subclonality.

**Fig. 2:**
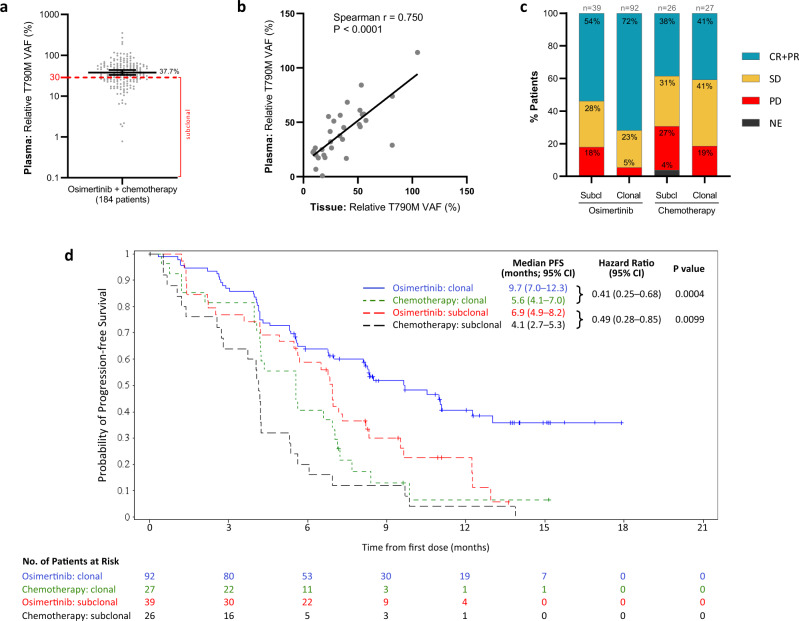
T790M clonality levels in baseline plasma and its association with tumour response and progression-free survival (PFS) on osimertinib and chemotherapy. **a** Distribution of relative T790M VAF in 184 selected AURA3. Black line represents the median (37.7%), ticks mark 95% CI (33.0–43.8%); red dashed line marks 30% T790M subclonality threshold. **b** Correlation between relative T790M VAF values calculated from plasma and tissue NGS data from 31 AURA3 patients for which both values were available. Spearman correlation = 0.750; *P* value = 0.0000012. **c** Patient’s best objective response (BOR) depending on the T790M subclonality group and treatment arm. BOR assessed according to Response Evaluation Criteria in Solid Tumours (RECIST). Relative T790M VAF of 30% was chosen as a subclonality threshold. CR + PR complete and partial response, SD stable disease, PD progressive disease, Subcl T790M subclonal, Clonal T790M clonal. **d** Kaplan–Meier estimates of the duration of PFS in subpopulations of patients with T790M subclonal and clonal tumours treated with osimertinib or chemotherapy. The tick marks indicate censored data. A hazard ratio <1 favours osimertinib. The HR, its two-sided 95% CI and *P* value are obtained from the unadjusted Cox proportional hazards.

The relative T790M VAF values were highly variable across patients with a median of 37.7% (95% CI: 33.0–43.8%) (Fig. 2a) and were not statistically significantly different between treatment arms (median of 40.9% for osimertinib and 31.1% for chemotherapy; Mann–Whitney test; *P* = 0.0606) (Supplementary Fig. 4a). We set a subclonality threshold below the median value and selected T790M VAF = 30% as a cut-off, which is in line with the range of previously published cutoffs^2,9,15,16^. This threshold enabled the inclusion of sufficient patient numbers in the T790M subclonal cohort, allowing us to perform powerful statistical analyses. Patients were then divided into the T790M subclonal (<30%) and T790M clonal (≥30%) groups in order to investigate the potential impact of subclonal levels of T790M on treatment outcome.

Out of the 184 patients with plasma relative T790M VAF value, 31 patients also had baseline tissue NGS data available. Reassuringly, the median tissue T790M clonality value (33.5%; 95% CI: 19.8–51.3%) (Supplementary Fig. 4b) was comparable to median plasma value (Fig. 2a) and the tissue and plasma T790M clonality levels were significantly correlated in these 31 patients (Spearman *r* = 0.750, *P* < 0.001) (Fig. 2b), with 25/31 (81%) samples showing concordance in the T790M clonality classification. The discrepant clonality result between plasma and tissue NGS in six samples is possibly associated with tumour heterogeneity that is better captured by plasma NGS.

The demographic characteristics of the patients at baseline were balanced between the two T790M clonality groups (Supplementary Tables 2 and 3). We next investigated the differences in tumour response, as defined by RECIST criteria, between the T790M clonality groups (Fig. 2c). Interestingly, the majority of osimertinib-treated patients with clonal T790M responded to the treatment (72% CR + PR), whereas the T790M subclonal cohort showed less responders (54% CR + PR) and more patients with SD and PD. No difference in the frequency of responders between the T790M clonality cohorts was observed in chemotherapy-treated patients, indicating that the association between tumour response and T790M subclonality could be treatment-specific.

In addition, assessment of the duration of progression-free survival (PFS) in the subclonal and clonal T790M cohorts revealed an association between T790M subclonality and poor PFS, both in the osimertinib (median PFS 6.9 months (95% CI: 4.9–8.2) in the subclonal vs 9.7 months (95% CI: 7.0–12.3) in the clonal group) and in the chemotherapy treatment arms (median PFS 4.1 months (95% CI: 2.7–5.3) in the subclonal group vs 5.6 months (95% CI: 4.1–7.0) in the clonal group) (Fig. 2d). Importantly, despite the different PFS between T790M subclonal and clonal groups in the osimertinib-treatment arm, both cohorts showed improved PFS when compared to the chemotherapy arm (subclonal groups: HR = 0.49 (95% CI: 0.28–0.85) and *P* = 0.0099; clonal groups: HR = 0.41 (95% CI: 0.25–0.68) and *P* = 0.0004 when comparing clonal groups), demonstrating the superiority of osimertinib over chemotherapy independently of the T790M clonality status.

### T790M subclonal group in osimertinib-treated patients is enriched for aberrations in the PIK3CA gene

**Fig. 3:**
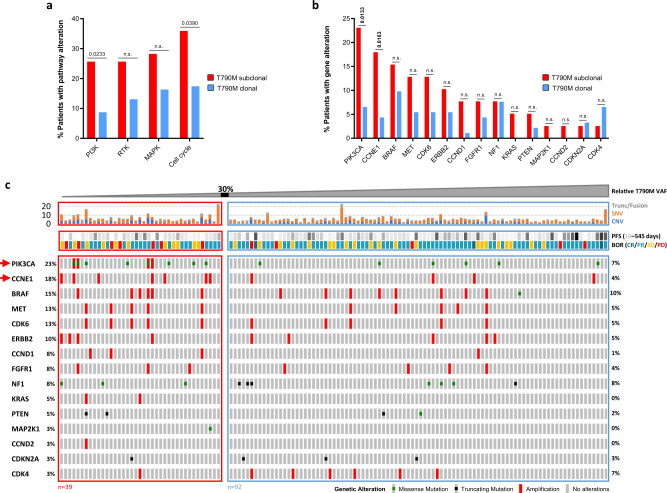
Genomic co-alterations detected in cfDNA of osimertinib-treated patients in each T790M clonality group. **a** Percentage of patients with functional alteration in selected pathways known to be involved in TKI resistance (list of pathway members included in the sequencing panel in Supplementary Table 4). Two-sided Fisher’s exact test was performed to detect statistically significant differences between the T790M subclonal (red, *n* = 39 patients) and clonal (blue, *n* = 92 patients) subgroups. N.s. not significant. **b** Percentage of patients with functional alteration(s) in selected genes in T790M subclonal (red) versus clonal (blue) subgroup. *P* value determined using the two-sided Fisher’s exact test. N.s. not significant. **c** Oncoprint of co-occurring mutation events in baseline plasmas and clinical response in osimertinib-treated patients with T790M clonality value (*n* = 131). Patients were sorted according to their relative T790M VAF value, the 30% subclonality threshold is marked. Only EGFR alterations and known pathogenic aberrations in genes known to be involved in TKI resistance are shown in the oncoprint. The top panel shows the number of all non-synonymous genetic aberrations (in all 73 tested genes) detected in each baseline plasma sample. Red arrows indicate genes significantly more frequently mutated in the T790M subclonal group. CNV events reported by Guardant Health for a copy number value >2. Trunc/Fusion truncating/fusion variants, SNV single-nucleotide variants, CNV copy number variants, PFS progression-free survival, BOR best objective response (assessed according to RECIST criteria), CR complete response, PR partial response, SD stable disease, PD progressive disease. Full list of gene alterations presented in the oncoprint is shown in Supplementary Table 5.

To understand the biology behind the worse clinical outcome of patients with T790M subclonal tumours to osimertinib, we aimed to characterise co-occurring genetic alterations in those tumours. We first investigated if the subclonal group showed enrichment for actionable alterations in signalling pathways known to be involved in resistance to tyrosine kinase inhibitors (TKIs), including members of the MAPK and PI3K pathways, cell cycle genes and receptor-tyrosine kinases (RTK), which could activate downstream signalling independently of the activating EGFR mutations (full gene list in Supplementary Table 4). Interestingly, the subclonal group had a significantly higher frequency of actionable alterations in the PI3K (Fisher’s exact test, *P* = 0.0233) and cell cycle pathways (Fisher’s exact test, *P* = 0.0390) (Fig. 3a). We then explored which genes within these pathways are significantly more frequently altered and identified that recurrent alterations in PIK3CA (*P* = 0.0133) and amplification in CCNE1 (*P* = 0.0163) have been significantly more frequently detected in the T790M subclonal compared to the clonal cohort (Fig. 3b, c). However, it is important to note that copy number alterations are challenging to detect and accurately quantify using plasma NGS and events may be missed^17^. The full list of gene alterations presented in Fig. 3c is shown in Supplementary Table 5.

We also investigated whether there is any difference in the number of detectable non-synonymous genomic aberrations between the T790M subclonality groups and found that the median number of aberrations was comparable between the groups (Supplementary Fig. 5).

### Distinct evolution of EGFR mutations between T790M subclonal and clonal groups in early time points after treatment with osimertinib

Because osimertinib targets both the EGFR-activating and T790M mutations, we next hypothesised that if other resistance-causing mutations are present at baseline, we would expect the elimination of the T790M cells, but the outgrowth of the non-T790M cells upon treatment with osimertinib. ddPCR was used to assess the dynamics of EGFR-mutant plasma ctDNA levels in early timepoints (baseline/week 3/week 6) after starting treatment with osimertinib. Data from at least two of the three studied timepoints were available for 104 patients, including 31 patients with subclonal and 73 patients with clonal T790M previously assessed by NGS. Despite the differences between the NGS and ddPCR assay platforms, a strong correlation between the assays was observed when determining VAFs for the ex19del/L858R and T790M mutations in baseline plasmas (Supplementary Fig. 6a, b).

**Fig. 4:**
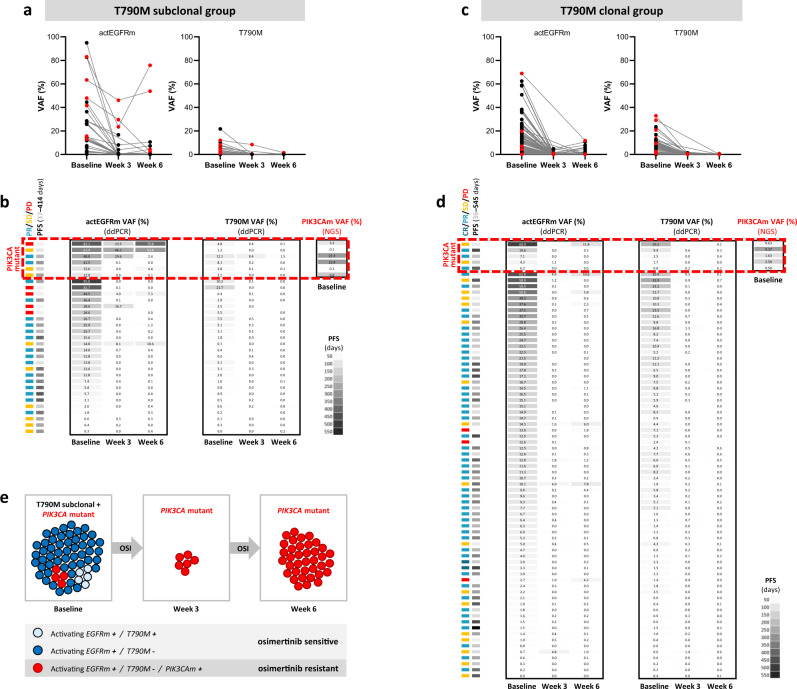
Changes in EGFR mutation shedding from baseline to week 6 in osimertinib-treated patients with clonal or subclonal T790M assessed by ddPCR. **a**, **c** Serial plasma ctDNA analysis of activating EGFR mutation (left) and EGFR T790M (right) shedding at baseline, week 3 and week 6 of treatment with osimertinib in 31 patients from the T790M subclonal cohort (**a**) and 73 patients from the T790M clonal cohort (**c**). The VAFs were determined by a ddPCR assay. Patients with PIK3CA mutant baseline plasma (detected by NGS) are highlighted in red. **b**, **d** Association of activating EGFR mutation (left) and EGFR T790M (right) VAF with clinical response in 31 patients from the T790M subclonal cohort (**b**) and 73 patients from the T790M clonal cohort (**d**). Patients with PIK3CA mutant baseline plasma (previously detected by NGS) are marked by a red rectangle and the highest detected VAF of actionable PIK3CA mutations in marked patients is shown. Tumour response (assessed according to RECIST criteria) categorised into CR complete response, PR partial response, SD stable disease, PD progressive disease, PFS progression-free survival. **e** A hypothetical schematic model of clonal evolution of the EGFRm + /T790M−/PIK3CAm + cell population (in red) from baseline until 6 weeks of treatment with osimertinib. Because osimertinib targets both the activating EGFRm and T790M, cells harbouring only these two driver mutations are quickly diminished by the treatment, whereas EGFRm + /T790M−/PIK3CAm + cells are expected not to respond to osimertinib that could drive resistance to osimertinib in these patients. OSI osimertinib.

As expected, osimertinib treatment led to a decrease in T790M levels in all patients from the T790M subclonal cohort (Fig. 4a, b) and a vast majority (71/73 patients, 97%) of patients from the T790M clonal group (Fig. 4c, d) after 3 weeks of treatment. The T790M VAFs further decreased or stayed below 1% in all patients after 6 weeks of treatment, independently on the T790M clonality status. Although the VAFs of the activating EGFR mutation were also decreased at weeks 3 and 6 when compared to baseline in both the T790M subclonal (Fig. 4a, b) and T790M clonal (Fig. 4c, d) groups, the VAF values were much more variable in the T790M subclonal group at both timepoints. These data suggest that a subpopulation of T790M-negative cells are more resistant to osimertinib treatment.

Because mutations in the PIK3CA gene were the most frequent genomic alterations detected in the T790M subclonal group by plasma NGS (Fig. 3b), we also explored changes in EGFR ctDNA shedding specifically in the limited number of PIK3CA mutant patients (highlighted in red in Fig. 4a, b). Interestingly, four out of six patients with PIK3CA mutant baseline plasma exhibited increased VAF of activating EGFR mutation from week 3 to week 6 (Fig. 4a, b), suggesting an outgrowth of the activating EGFRm+/T790M− clone and a potential presence of the PIK3CA mutation that could drive resistance to osimertinib in these patients (Fig. 4e). In addition, one out of four evaluable PIK3CA mutant patients from the clonal group showed an increased activating EGFR mutation VAF from week 3 to week 6 (Fig. 4c, d), indicating that PIK3CA mutation could also co-occur with T790M.

### PIK3CA H1047R mutation drives resistance to osimertinib in vitro, which can be overcome by combination treatment with PI3K pathway inhibitors

In order to test the hypothesis that a PIK3CA-activating mutation drives resistance to osimertinib, we introduced the PIK3CA H1047R (hereafter referred to as PIK3CAm) variant in three lung cancer cell line models using a CRISPR/Cas9 technology: PC9, PC9-T790M and HCC827. As the efficiency of knock-ins using this technology is usually low, only a small proportion of cells (a subclone) was expected to be genetically modified^18^, thus mimicking the emergence of a co-occurring resistance mutation in a tumour. This heterogeneous cell pool was then cultured under the selection pressure of osimertinib for 2–3 weeks to generate an osimertinib-resistant cell pool for downstream analyses (Supplementary Fig. 7a).

**Fig. 5:**
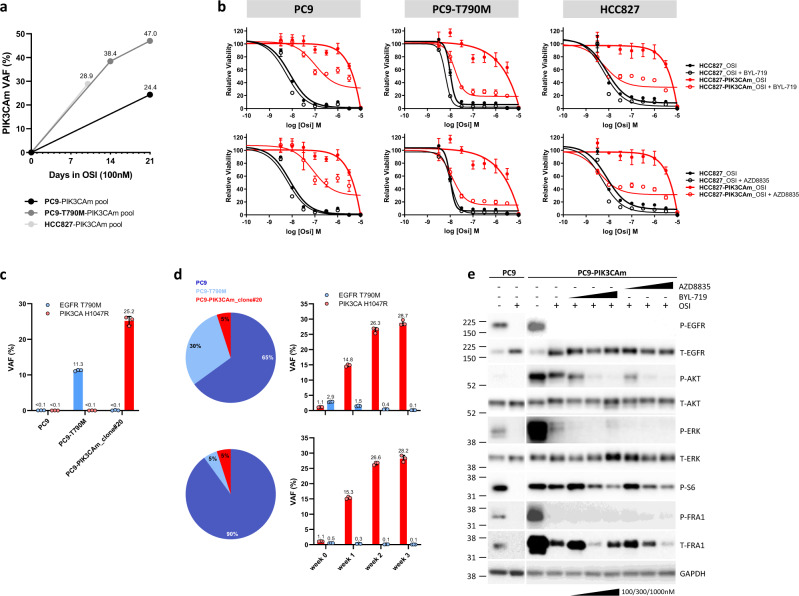
In vitro functional analysis of the PIK3CA H1047R mutation (PIK3CAm) detected in multiple baseline plasmas from AURA3 patients. **a** Dynamics of PIK3CA H1047R VAF changes in individual CRISPRed cell lines treated with 100 nM osimertinib for 2–3 weeks. The HCC827–PIK3CAm pool was treated for 2 weeks (DNA analysis at day 10), the other two cell line pools were treated for 3 weeks. DNA from PC9–PIK3CAm pool was assessed by NGS, the other two cell line pools were genotyped by ddPCR. PIK3CAm refers to PIK3CA H1047R. **b** Effect of osimertinib and 500 nM PI3K pathway inhibitor (BYL-719, AZD8835) co-treatment in the PIK3CA H1047R mutant cell pool and PIK3CA WT parental cell lines grown in 2D monolayer. Parental cell lines PC9, PC9-T790M and HCC827 were tested. Representative experiments from three independent repeats are shown, error bars represent mean ± SD from replicate wells. **c** EGFR T790M and PIK3CA H1047R genotyping in three cell line models used in a co-culture experiment: PC9, PC9-T790M (EGFR T790M VAF = 11.3 ±0.212%), PC9-PIK3CAm_clone#20 (PIK3CA H1047R VAF = 25.2 ± 1.272%). Black lines and error bars represent the mean ± SD VAF (%) from three independent ddPCR runs with optimised conditions for each ddPCR assay. The mean VAFs where values are not specified were <0.1%. **d** Pie charts (left panel) show the percentage of each cell line model in each of the two co-cultures tested in this experiment. The dynamics of EGFR T790M and PIK3CA H1047R VAF changes between weeks 0–3 of treatment with 160 nM osimertinib in each co-culture is shown in the bar charts (right panel). Values and error bars represent the mean ± SD VAF (%) from three independent experiments using optimised ddPCR assays. **e** Immunoblot analysis of PC9 parental cells and PC9–PIK3CA–H1047R CRISPR cell pool grown in 2D monolayer and treated with vehicle (DMSO, marked as −), osimertinib (OSI) alone or with a combination with BYL-719 or AZD8853. Treatment concentrations were the following: 100 nM OSI, 100 nM/300 nM/1000 nM BYL-719 or AZD8853. Immunoblot analysis was performed on a cellular extract of cells treated for 4 h by indicated compounds and concentrations. Immunoblotting analysis has been repeated at least once for each treatment condition. Source data are provided as a Source Data file.

Indeed, long-term treatment of the CRISPR cell pool with osimertinib led to a selective outgrowth of the PIK3CAm-positive cells in all three cell lines (Fig. 5a and Supplementary Fig. 7b), indicating that the mutation conferred resistance to osimertinib. To assess the PIK3CAm-induced resistance in more detail, the viability of the osimertinib-resistant PC9–PIK3CAm cell pool was compared to the parental PC9 cells upon treatment with osimertinib alone or in combination with inhibitors of the PI3K pathway directly targeting the PI3Kα subunit (BYL-719 or AZD8835) for 6 days, showing that the PIK3CAm-positive cells were resistant to osimertinib in both 2D monolayer (Fig. 5b) and 3D spheroid cell cultures (Supplementary Fig. 7c). Importantly, co-treatment with PI3K pathway inhibitors was able to partially re-sensitise cells to osimertinib in both 2D (Fig. 5b) and 3D cultures (Supplementary Fig. 7c). Similar findings were observed in the PC9-T790M and HCC827 cell line models, where knock-in of the PIK3CAm resulted in resistance to osimertinib, which could be partially rescued by a co-treatment with PI3K pathway inhibitors (Fig. 5b), offering possible therapeutic strategies to combat TKI resistance driven by PIK3CA mutations.

To further model tumour heterogeneity and assess the dynamics of response of various cell clones to osimertinib, we performed a long-term co-culture experiment by mixing the PC9 cells with two different subclonal levels of PC9-T790M cells and PC9–PIK3CAm cell clone and treated the co-cultures with osimertinib for up to 3 weeks. EGFR T790M and PIK3CA H1047R VAFs were assessed in each individual cell line model (Fig. 5c) used for the co-culture experiments and in both co-cultures over time (Fig. 5d). Osimertinib treatment rapidly selected for the outgrowth of the PIK3CA mutant cell clone. This selective outgrowth was independent of the size of the T790M mutant clone that became almost undetectable after 2–3 weeks of treatment (Fig. 5d).

Protein analysis by western blot showed an increase in the basal level of pAKT, pERK and pS6 in the PC9–PIK3CAm CRISPR cell pool when compared to parental PC9 cells, indicating activation of downstream PI3K/AKT and MAPK signalling pathways in the osimertinib-resistant cells (Fig. 5e). Treatment with osimertinib resulted in downregulation of P-EGFR levels and MAPK signalling in both parental and PC9–PIK3CAm cells. However, the PC9–PIK3CAm cells displayed only partial inhibition of the PI3K/AKT signalling pathway in the presence of osimertinib, indicating that the PIK3CA mutation leads to a resistance to osimertinib through the activated PI3K pathway, but not through the MAPK signalling cascade. Of note, co-treatment of PC9–PIK3CAm cells with osimertinib and PI3K pathway inhibitor (BYL-719 or AZD8835) resulted in further downregulation of AKT signalling in a dose-dependent manner (Fig. 5e).

## Discussion

Genomic diversity and the presence of multiple subclones within single tumours are key challenges in the treatment of cancer patients with targeted therapies. Some evidence suggests that T790M subclonality, i.e. the presence of EGFR T790M-positive cells in only a subset of tumour cells, might influence the response of NSCLC patients to the third-generation TKI osimertinib^9,11^. Given that osimertinib is now approved for the second-line treatment of EGFR-mutant advanced NSCLC patients who progressed on previous lines of TKI, it is critical to elucidate the role of T790M subclonality in response to this agent. In this retrospective analysis of baseline plasma samples from patients enrolled in the AURA3 study^12^, we showed that T790M subclonality is associated with worse clinical outcome in osimertinib-treated patients possibly through the presence of other co-occurring TKI-resistance alterations, in particular in the PIK3CA gene. Our in vitro data showed that activating PIK3CA mutation gives cells a growth advantage under osimertinib selection pressure through activation of the PI3K pathway, which could potentially lead to osimertinib resistance in a clinical setting^19^. This PIK3CA mutant-driven resistance could be overcome by combination treatment with PI3K pathway inhibitors in vitro, providing a possible rationale for a combination treatment.

There is no strict definition of a ‘subclonal’ mutation and studies to date have defined such mutations as alterations being present in less than 10–50% frequency in the tumour^2,9,15,16,20–22^. Because we were interested to study the effect of a low fraction of T790M, we set the subclonality threshold below the median T790M subclonality value (median = 37.7%, Fig. 2a) and selected 30% as a cut-off. A more extreme threshold would not allow the inclusion of sufficient patient numbers in the T790M subclonal cohort and we would not be able to perform meaningful statistical analyses.

The assessment of clinical outcome in the T790M subclonal vs clonal cohorts revealed an association between subclonal T790M and poor PFS, both in the osimertinib and in the chemotherapy treatment arms (Fig. 2d). Considering chemotherapy is presumed to have a relatively non-selective cytotoxic effect on all rapidly dividing cells, whereas osimertinib acts against activating and resistance EGFR mutations^7^, the association of T790M subclonality with shorter PFS on both treatments could be influenced by other, non-T790M alterations co-occurring in these tumours. In fact, although EGFR T790M is the most common resistance mechanism to first- and second-generation TKIs, resistance mutations in genes such as MET, PIK3CA, NF1, are also often found and these can be present in distinct cell populations from T790M^2^.

It is important to note that the patients with subclonal T790M still benefit from treatment with osimertinib compared to chemotherapy, but our data suggest that patients from the subclonal group develop resistance to the osimertinib treatment more rapidly than patients with a clonal presence of T790M. This could be caused by the mixed sensitivities of distinct cellular clones to osimertinib. In fact, our data from early timepoints longitudinal analysis show that osimertinib is able to suppress the activating EGFRm-positive/T790M-positive clone, but the activating EGFRm-positive/T790M-negative clone showed resistance to the treatment in a subgroup of patients as was evidenced by a less significant drop in ctDNA levels at week 3 (Fig. 4a, b). As osimertinib acts against both activating EGFR mutations and T790M^7^, observed resistance of the activating EGFR mutation-positive clone could be due to a presence of other mechanisms of TKI resistance in the cells that do not harbour the T790M mutation^2,14,23^. Outgrowth of a TKI-resistant clone after clearance of the T790M-positive subclone is in line with the previously observed association between the loss of T790M mutation at progression and shorter mPFS in NSCLC patients^11,24^.

Genes in the PI3K and cell cycle pathways, in particular PIK3CA and CCNE1, were the most frequently altered TKI-resistance genes in the T790M subclonal cohort. We focused our attention on the PI3K pathway alterations as these are targetable with inhibitors of the PI3K/AKT/mTOR signalling cascade^25–27^. Clinically actionable PIK3CA mutations in exons 9 and 20 have been previously found in the EGFR-mutant tumours of patients with acquired resistance to first- and second-generation EGFR-TKIs^5,28–30^ and could have been selected as a result of TKI selective pressure. In fact, PIK3CA gene alterations have been consistently one of the most frequently co-occurring known TKI-resistance alterations in T790M-positive advanced NSCLC^2,31,32^. However, in vitro evidence that these mutations confer resistance to osimertinib is limited. To our knowledge, our results from PIK3CA H1047R CRISPR knock-in and co-culture experiments provide the first in vitro demonstration that PIK3CA H1047R emergence in a small fraction of cells gives these cells a growth advantage over the PIK3CA WT clone under osimertinib treatment, independently of whether the PIK3CA mutation occurs in a EGFR T790M mutant or T790 WT cell. These results show that subclonal levels of PIK3CA mutations, which could be acquired during previous lines of therapies with first/second generation of EGFR-TKIs in patients with advanced-stage EGFR-mutant NSCLC, might have important therapeutic implications. The subclonal presence of EGFR-TKI-resistance alterations raises the question of whether these mutations would be detected in a tumour biopsy. With the caveat of small numbers, our initial plasma vs tumour tissue analysis showed a good concordance, and thus a non-invasive ctDNA analysis from plasma liquid biopsy seems to be an ideal option to screen for known EGFR-TKI-resistance mutations and could inform the selection of potential personalised treatment combinations.

In addition, our in vitro experiments from multiple cell line models also indicate that PIK3CA H1047R-mediated resistance to osimertinib could be, at least partially, reversed by co-treatment with PI3K pathway inhibitors. These observations warrant further investigations into a potential combination treatment with osimertinib and an inhibitor of the PI3K pathway and more detailed comparison of the most effective osimertinib combination partner as multiple compounds targeting the PI3K pathway are entering the clinic, or are under investigation in registrational clinical studies^33–35^. In addition, our results may also have important implication for the first-line osimertinib treatment setting, as PIK3CA mutations have been described to be acquired after the first-line osimertinib in the FLAURA phase III study^36–38^. Thus, future ctDNA plasma testing and identification of PI3K pathway alteration in EGFR-mutant lung cancer patients could classify the patient for a combination treatment of osimertinib and PI3K pathway inhibitor. Furthermore, detection of T790M subclonality in pre-treatment plasmas from advanced NSCLC patients, for example by the comprehensive liquid biopsy Guardant360 assay which currently reports mutation clonality status in a research setting^20^, could lead to a more frequent liquid biopsy monitoring of the patients to detect expanding resistant clones early, before the occurrence of clinical and radiographic changes.

We are aware of certain limitations of our study. One of the key enrolment criteria into the AURA3 trial was a centrally confirmed T790M-positive tumour tissue after first-line EGFR-TKI therapy. Because advanced-stage NSCLC tumours are genomically heterogeneous and the T790M-positive and negative cell populations can co-occur^2^, it is probable that a fraction of T790M-positive tumours was missed by the central test, especially if T790M was extremely subclonal and tissue biopsy could have easily missed such a small subclonal cell population. It is therefore likely that the extreme T790M subclonal cohort is underrepresented in this study set, which makes the evaluation of the impact of T790M subclonality on response to osimertinib more challenging. In addition, 36% of patients could not be included in the T790M subclonality analysis because their plasma was ctDNA-negative for either the activating or resistance EGFR mutations or for both mutations (EGFR nonshedders), hampering a robust assessment of T790M subclonality levels. A recent publication from our collaborators shows that EGFR nonshedders have smaller baseline tumour target lesion size^39^, being in line with our observation that this cohort has the least number of detectable genomic alterations and the lowest VAFs of these mutations (Supplementary Fig. 2b, c). In addition, the analysis of tissue and/or liquid biopsy samples from patients, who have progressed on osimertinib, would help us to study the dynamics and implications of clonal evolution of actionable PIK3CA mutations.

Overall, our results shed light on the implications of T790M subclonality in resistance to osimertinib in advanced-stage NSCLC patients and highlight the potential of using ctDNA mutation analysis to identify these patients. In addition, we demonstrated a higher detection rate of PIK3CA mutations in those patients and show that co-occurring PIK3CA mutations drive resistance to osimertinib in vitro, which has the potential to be overcome by co-treatment with an inhibitor of the PI3K pathway in the clinical setting.

## Methods

### Patients and plasma ctDNA analysis

We retrospectively evaluated plasma samples collected at baseline from 289 patients enrolled into the AURA3 study (ClinicalTrials.gov trial registration no. NCT02151981). All the patients provided written informed consent before the screening and we have complied with all relevant ethical regulations. The study was performed in accordance with ethical principles that have their origin in the Declaration of Helsinki and are consistent with ICH/Good Clinical Practice, applicable regulatory requirements and the AstraZeneca policy on Bioethics and Human Biological Samples. Full details of the methodology of the AURA3 study have been published previously^12^. Briefly, patients randomised to treatment provided blood samples for plasma cell-free DNA (cfDNA) analysis at baseline (prior to the first dose of study drug) and at weeks three and six on treatment. Plasma cfDNA was analysed for genetic alterations in 73 genes using the Guardant360 assay (Guardant Health, CA, USA), a commercially available next-generation sequencing (NGS) test (Supplementary Table 1). In addition, plasma samples collected at baseline, three and six weeks on treatment, were analysed for EGFR mutations (T790M, exon 19 deletion (ex19del), L858R) using a droplet digital polymerase chain reaction (ddPCR) assay (Biodesix, CO, USA)^40^. Frequencies of genomic alterations detected in a given sample by NGS or ddPCR are shown as variant allele frequency (VAF), i.e. the percentage of sequence reads observed matching a specific DNA variant divided by the overall coverage at that locus.

Only patients with detectable EGFR driver mutation (ex19del or L858R) as well as resistance T790M mutation in baseline plasma were included in the calculation of T790M subclonality levels. T790M subclonality (i.e. relative T790M VAF) has been calculated as a ratio of the EGFR T790M VAF over the activating EGFR mutation (L858R, exon 19 deletion) VAF, presented as a percentage. T790M mutation was considered as subclonal if the relative T790M VAF value was lower than 30%.

### Tumour tissue NGS

Tumour NGS was performed retrospectively on tumour tissue samples from 31 AURA3 patients using the FoundationOne CDx panel^41^. T790M subclonality (i.e. relative T790M VAF) has been calculated as a ratio of the EGFR T790M VAF over the activating EGFR mutation (L858R, exon 19 deletion) VAF, presented as a percentage.

### Cell lines and reagents

The PC9 (EGFR ex19del E746-A750del) and HCC827 (EGFR ex19del E746-A750del) cell lines were obtained, authenticated and cultured as recommended by the European Collection of Authenticated Cell Cultures (ECACC) and the American Type Culture Collection (ATCC). The PC9-T790M (EGFR ex19del E746-A750del, EGFR T790M) cells were obtained by treatment of PC9 cells with 1.5 μM Iressa for 4 weeks. Acquired EGFR T790M mutation was confirmed by ddPCR as described in Methods. The cell lines were confirmed to be negative for mycoplasma. All cell lines were cultured in RPMI 1640 medium (Gibco) supplemented with 10% FBS (Gibco) and were grown at 37 °C, in a humidified atmosphere with 5% CO_2_. Osimertinib, AZD8835, BYL-719 and Iressa were synthesised at AstraZeneca according to published methods^42–45^.

### CRISPR-based knock-in (KI) of PIK3CA H1047R into lung cancer cell models

CRISPR/Cas9 technology has been used in order to knock-in (KI) PIK3CA H1047R mutation into the PC9, PC9-T790M and HCC827 cellular models. Briefly, cells were electroporated at 1400 V, 20 ms, two pulses (Neon Transfection System, ThermoFisher Scientific) with Cas9 recombinant protein complexed with Alt-R^®^ CRISPR–Cas9 tracrRNA and Alt-R^®^ CRISPR–Cas9 sgRNA (Integrated DNA Technologies, Sigma) with sequence 5′-CAAATGAATGATGCACATCA-3′ in conjunction with a synthetic single-strand DNA oligo donor (Ultramer oligo, Integrated DNA Technologies, Sigma) with homology arms to PIK3CA harbouring the H1047R mutation and the following sequence: AAACTGAGCAAGAGGCTTTGGAGTATTTCATGAAACAAATGAATGATGCACGTCATGGTGGCTGGACAACAAAAATGGATTGGATCTTCCACACAATTAAACA. Transfected cells were positively selected with 100 nM osimertinib for 2–3 weeks (2 weeks for HCC827; 3 weeks for PC9, PC9-T790M) prior to expansion.

The single-cell PC9–PIK3CA-H1047R clone #20 was generated by limiting dilution from the osimertinib-resistant PC9–PIK3CA-H1047R CRISPR cell pool and PIK3CA H1047R was confirmed by ddPCR as described in Methods.

### Next-generation sequencing of PIK3CA locus and bioinformatics

Genomic DNA was isolated from cell pellets using the DNeasy Blood & Tissue Kit (Qiagen) according to the manufacturer’s instructions. In all, 12.5 ng of genomic DNA were amplified using a two-step PCR that added unique library barcodes, heterogeneity spacers and Illumina MiSeq adapters^46,47^. Primer sequences for two-step PCR are shown in Supplementary Table 6. Samples were sequenced using a MiSeq^®^ Reagent Kit v2 (300 cycles) (Illumina) on a MiSeq instrument (Illumina). Quantification and classification of the sequences were done using the following tools: Fast Length Adjustment of Short reads (FLASH v1.2.11) was used to group paired reads. BWA-MEM was used to align to the human genome (hg19) or the BFP coding sequence. Samtools was used to generate sorted, indexed BAM files. Samtools was used to generate data for variant calling with the following options: minimum read depth 1000, minimum quality 25, minimum allele frequency 0.005 (0.5%), maximum mismatch 100 and trim 20^48^. Amplicon sequencing summary is presented in Supplementary Table 7.

### EGFR T790M and PIK3CA H1047R genotyping by ddPCR

Genomic DNA was isolated from cell pellets using the DNeasy Blood & Tissue Kit (Qiagen) according to the manufacturer’s instructions. Reaction volumes were made up to 20 μl and partitioned to up to 20,000 droplets using a ddPCR Auto Droplet Generator (Bio-Rad). For mutation analysis, the following conditions were used: 95 °C for 10 min followed by 40 cycles of 94 °C for 30 s, then 57.3 °C for 60 s, ramp rate 2 °C/s, 98 °C for 10 min and final incubation 12 °C for at least 4 h. The subsequent analysis was done on a Bio-Rad QX200 droplet reader and analysed using QX Manager Software Standard Edition v1.1 (Bio-Rad). EGFR T790M and PIK3CA H1047R primer/probe sequences are shown in Supplementary Table 6.

### Cell viability and growth assays

For 2D assays, cells were plated at 2000 cells/well in 96-well and osimertinib was added to plates using the HP D300 digital dispenser in a ½ log dilution (10 µM to 0.003 µM). 500 nM of BYL-719 and AZD8835 were added where indicated. Compound volumes were normalised with DMSO and control wells were filled with DMSO. Treated cells were cultured for 5 days and analysed for cell viability using CellTiter-Glo assay (Promega) as per the manufacturer’s instructions. Each plate was normalised to vehicle control and data were analysed using GraphPad Prism. Each condition was run in triplicate 96 wells, graphs show representative results from three independent experiments.

For 3D assays, 1000 cells/well were seeded into a 96-well clear round-bottom ultra-low-attachment microplate (Corning) and cultured for 3 days in RPMI 1640 supplemented with 1% phenol red-free matrigel (Corning) and 2% FBS. Cells were then supplemented with additional FBS to a total concentration of 10% and treated with DMSO (control), osimertinib (0.1–100 nM), BYL-719 (500 nM) or AZD8835 (500 nM) for 6 days. Cell viability was assessed using CellTiter-Glo assay (Promega) as per the manufacturer’s instructions and luminescence was read on the Envision (PerkinElmer) instrument. Data were exported to GraphPad Prism and the values normalised to the largest mean in each dataset and background control. Each condition was run in duplicate wells, graphs show representative results from three independent experiments.

### Co-culture experiment

Co-culture of cell lines PC9: PC9-T790M: PC9–PIK3CA-H1047R-clone#20 was prepared in the following ratios 65:30:5 or 90:5:5 with the final seeding density of each co-culture of 3 × 10^5^ cells. Cells were cultured in RPMI 1640 medium (Gibco) supplemented with 10% FBS (Gibco) in the presence of 160 nM osimertinib. The cell pellet was collected at days 0, 7, 14, 21 of the treatment, and genomic DNA was isolated and genotyped by ddPCR as described in ‘Methods’.

### Immunoblotting

Cells were lysed in RIPA buffer (ThermoFisher), supplemented with protease inhibitor cocktail (Merck Millipore 539131-1VL), phosphatase inhibitor cocktail II (Merck Millipore 524625-1set). Primary antibodies: Phospho-AKT S473 (1:1000; CST cat. no. 4060), AKT (1:1000; CST cat. no. 9272), phospho ERK T202/Y204 (1:1000, CST cat. no. 9101), ERK1/2 (1:1000; CST cat. no. 9102), phospho ribosomal protein S6 S235/236 (1:1000; CST cat. no. 4858), phospho FRA1 (1:1000; CST cat. no. 3880), FRA1 (1:1000; CST cat. no. 5281), phospho EGFR (1:1000; CST cat. no. 2234), EGFR (1:1000; CST cat. no. 4267), GAPDH (1:2000; CST cat. no. 5174). Anti-mouse and anti-rabbit IgG secondary antibodies were coupled to horseradish peroxidase (1:2000; CST). All full scan blots are presented in supplied Source data files uploaded to Nature Communications.

### Statistics

Unless otherwise indicated, the graphs have been made using the GraphPad Prism software (versions 8.0.1, 9.0.0) and the descriptive statistics and statistical tests were conducted using the SAS software (version 9.2). Mann–Whitney test was used to compare differences between two independent groups when the data were not normally distributed. Spearman correlation was used to correlate data that were nonnormally distributed. To compare two dependent groups in Supplementary Fig. 1, a random-effect model was fitted to log10(VAF) separately for each of the above two comparisons, by including mutation type as a fixed effect and patient as a random effect in the model. The two-sided *P* values corresponding to testing for a difference in the log10(VAF) LS-means between the mutation types were presented. To determine differences in the frequency of genomic alterations between cohorts (T790M subclonal versus clonal), we used two-tailed Fisher’s exact tests. Co-occurring genomic alterations have been visualised in an oncoprint figure using the Oncoprinter tool (cBioPortal, https://www.cbioportal.org/oncoprinter). For assessments of PFS, the 95% CI for the median duration of PFS was calculated using the Kaplan–Meier method. A hazard ratio (HR) < 1 favours osimertinib. The HR, its two-sided 95% CI and *P* value are obtained from the unadjusted Cox proportional hazards model (using profile likelihood CIs and Efron approach for handling ties) and the Kaplan–Meier plot was generated using SAS.

### Reporting summary

Further information on research design is available in the Nature Research Reporting Summary linked to this article.

## Supplementary information

Supplementary Information

Reporting Summary

## Data Availability

The 73-gene panel analysed by NGS-based cfDNA Guardant360 assay is reported in Supplementary Table 1, the gene alterations listed in Supplementary Table 5 and patient demographics data in Supplementary Tables 2 and 3. The raw sequencing data are not publicly available due to data privacy regulations and restrictions for use of such data as stated in the study protocol and patient consent form. Individual-level data can potentially be accessed via a collaborative agreement with AstraZeneca Group. The authors declare that the clinical dataset analysed here, including progression-free survival and tumour response data, is available and may be obtained in accordance with AstraZeneca’s data sharing policy as part of an external collaborative request (https://astrazenecagroup-dt.pharmacm.com//DT/Home/Index/) or an external data access request (https://vivli.org/ourmember/astrazeneca/). A reporting summary for this article is available as a Supplementary Information file. All remaining data are available within the article supplementary information or available from the authors upon reasonable request. Source data are provided with this paper.

## Source data

Source Data
