# Supplementary material for: Clinical impact of subclonal EGFR T790M mutations in advanced-stage EGFR-mutant non-small-cell lung cancers
Source: Nat Commun. 2021 Mar 19;12:1780. doi: 10.1038/s41467-021-22057-8 (PMC7979775; doi:10.1038/s41467-021-22057-8)
Supplement: Supplementary file 1 — Supplementary Information [file 41467_2021_22057_MOESM1_ESM.pdf]

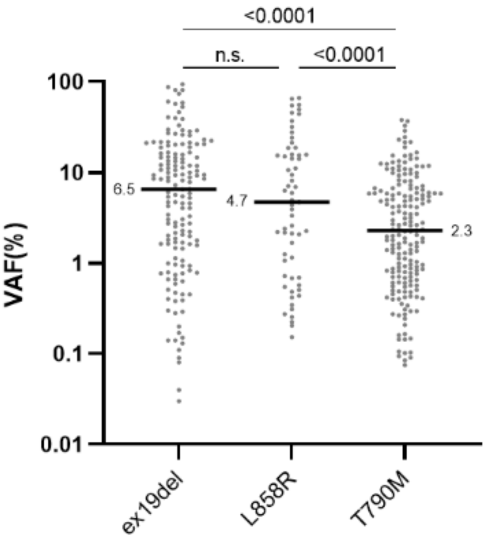

Supplementary Fig. 1: Distribution of EGFR ex19del, L858R or T790M VAFs detectable in baseline plasma cfDNA by next-generation sequencing. Lines represent the median VAF values. P value for comparison between ex19del and L858R VAF values was determined by Mann Whitney test. n.s., not significant (P=0.6687). Different method has been used for the remaining two comparisons, ex19del versus T790M VAFs and L858R versus T790M VAFs, due to group dependences. A random effects model was fitted to log10(VAF) separately for each of the above 2 comparisons, by including mutation type as a fixed effect and patient as a random effect in the model. The two-sided p-values corresponding to testing for a difference in the log10(VAF) LS-means between the mutation types are presented. The normality assumptions of the model were also met.

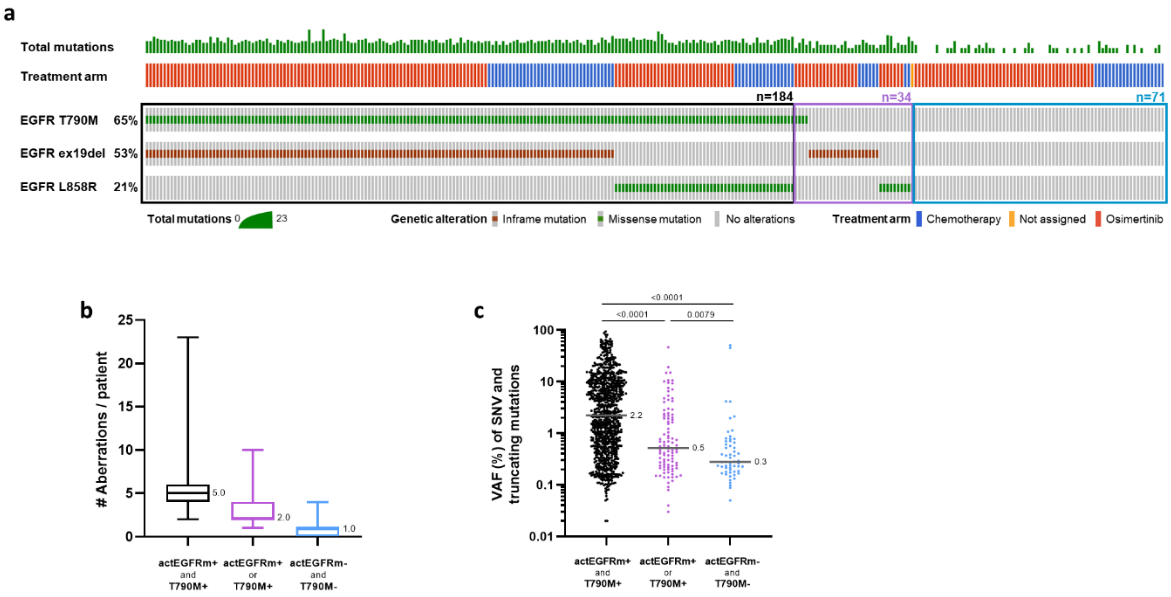

Supplementary Fig. 2: Analysis of ctDNA mutation shedding by baseline plasma NGS among different EGFR mutation shedding groups. (a) Oncoprint showing concurrent EGFR alterations detected in 289 AURA3 patients. A total number of detectable alterations per patient is shown in a histogram at the top of the oncoprint. Three EGFR shedding groups are specified: Patients positive for both activating EGFR mutation (actEGFRm) and T790M are marked by a black rectangle, patients with only actEGFRm or T790M detectable in baseline plasma are marked by a purple rectangle, patients with none of the EGFR mutations (EGFR nonshedders) are marked by a blue rectangle. (b) Median number of detectable genomic aberrations (SNVs, indels, fusions, amplifications) in the 73 tested genes per patient in the three EGFR shedding groups. The box-whisker graph shows a minimum (upper whisker), maximum (lower whisker), median (middle line; value shown) and 25th and 75th percentiles (box). (c) Median VAF values of non-synonymous SNVs and truncating mutations (indels, fusions) detected in patients from the three EGFR shedding groups. 37 out of 71 (52%) EGFR non-shedders had at least 1 detectable non-synonymous SNV, indel or fusion. Line

represents the median VAF of detectable aberrations. P value was determined by the Mann Whitney test, two-sided.

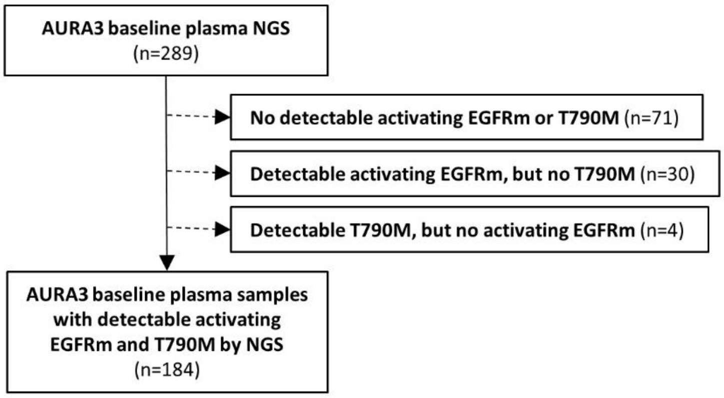

Supplementary Fig. 3: Patient plasma sample selection for T790M subclonality analysis. Patients from both treatment arms, osimertinib 80mg and chemotherapy, have been included in the T790M subclonality evaluation.

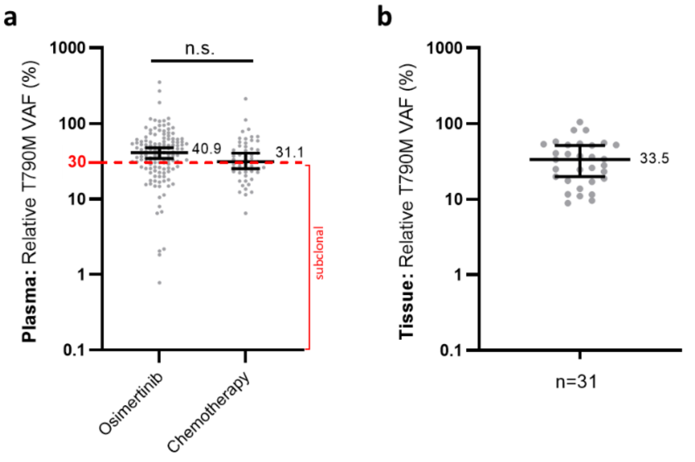

Supplementary Fig. 4: Distribution of relative T790M VAF in plasma and tumour samples. (a) Relative T790M VAF values in a selection of 131 osimertinib-treated and 53 chemotherapy-treated patients. The median relative T790M VAF value is 40.9% and 31.1% in the osimertinib and chemotherapy treatment arms, respectively. Black line represents median, ticks mark 95% CI; red dashed line marks the 30% T790M subclonality threshold. P value determined by Mann Whitney test, P=0.0606; n.s., not significant. (b) Distribution of relative T790M VAF in 31 tumour (=tissue) samples. The relative T790M VAF values were calculated as described for plasma (details in Methods). Black line represents median (33.5%), ticks mark 95% CI.

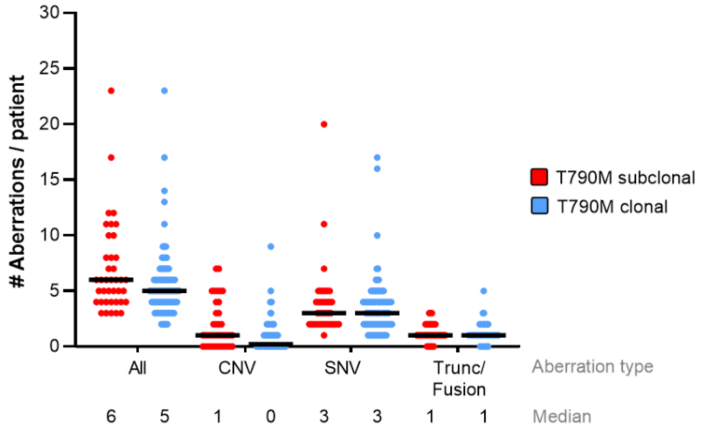

Supplementary Fig. 5: Number of non-synonymous aberrations detected in baseline plasmas by a cfDNA assay in T790M subclonal (red) and clonal (blue) groups. CNV, copy number variants; SNV, single-nucleotide variants; Trunc/Fusion, truncating/fusion variants. Among the CNV category, only amplifications have been detected using the clinically validated NGS-based cfDNA assay. Black line indicates median.

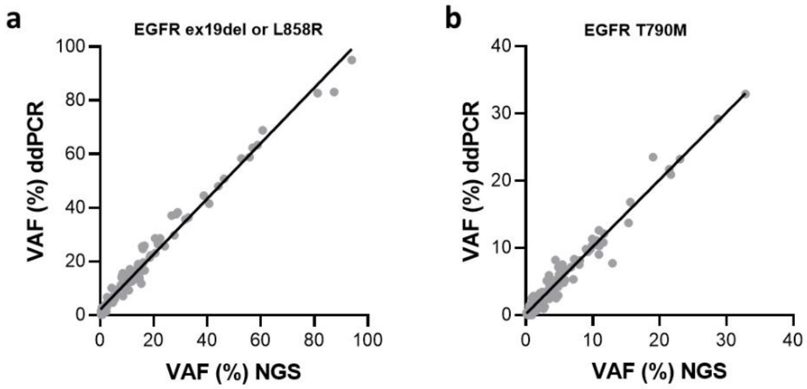

Supplementary Fig. 6: Correlation between EGFR mutation VAFs detected in baseline plasma by the ddPCR and NGS assay platforms. Values for the EGFR ex19del/L858R (a) and the EGFR T790M VAF (b) obtained by the two assay platforms are plotted. Data from 104 AURA3 patients assessed in Figure 4 were included (patients with ddPCR data from at least two of the three studied timepoints, including 31 patients with subclonal and 73 patients with clonal T790M previously assessed by NGS).

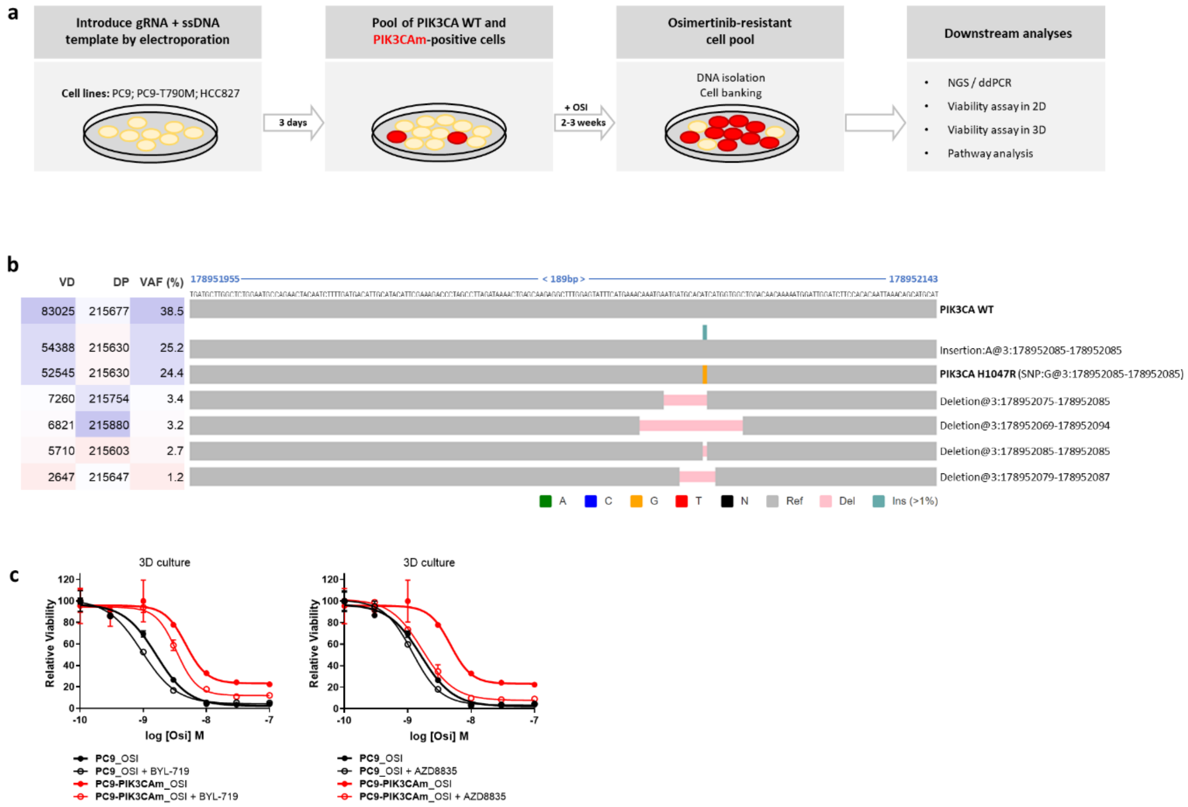

Supplementary Fig. 7: In vitro validation of PIK3CA H1047R as a driver of resistance to osimertinib in EGFR-mutant lung cancer cell lines. (a) CRISPR/Cas9-mediated genomic knock-in (KI) of PIK3CA H1047R to understand its contribution as a driver of resistance to osimertinib (details in the Methods section). (b) Frequency of individual variants in a PIK3CA amplicon detected by NGS of the PC9-PIK3CA-H1047R CRISPR cell pool after 3 weeks of osimertinib treatment. NGS revealed PIK3CA H1047R at VAF=24.4% and numerous small insertions/deletions, a common feature of CRISPR knock-in experiment due to a high rate of an imprecise repair by the non-homologous end joining (NHEJ) pathway. Only variants with VAF>1% are shown. VD, variant depth; DP, read depth; VAF (%), variant allele frequency [%]; Ref, reference sequence; Del, deletion; Ins, insertion. (c) Effect of osimertinib and 500nM PI3K inhibitor (BYL-719 on the left, AZD8835 on the right) co-

treatment in the PIK3CA H1047R mutant cell pool and WT PC9 cells grown in 3D spheroid culture. Representative experiments from three independent repeats are shown, error bars represent mean ± SD from replicate wells. Source data are provided as a Source Data file.

Supplementary Table 1: Guardant360 sequencing panel (G360; 73 genes).

Gene	SNVs	Indels	Amplifications	Fusions
AKT1	yes			
ALK	yes			yes
APC	yes	yes		
AR	yes		yes	
ARAF	yes			
ARID1A	yes	yes		
ATM	yes	yes		
BRAF	yes		yes	
BRCA1	yes	yes		
BRCA2	yes	yes		
CCND1	yes		yes	
CCND2	yes		yes	
CCNE1	yes		yes	
CDH1	yes	yes		
CDK4	yes		yes	
CDK6	yes		yes	
CDKN2A	yes	yes		
CTNNB1	yes			
DDR2	yes			
EGFR	yes	yes	yes	
ERBB2	yes	yes	yes	
ESR1	yes			
EZH2	yes			
FBXW7	yes			
FGFR1	yes		yes	
FGFR2	yes		yes	yes
FGFR3	yes			yes
GATA3	yes	yes		
GNA11	yes			
GNAQ	yes			
GNAS	yes			
HNF1A	yes			
HRAS	yes			
IDH1	yes			
IDH2	yes			
JAK2	yes			
JAK3	yes			
KIT	yes	yes	yes	
KRAS	yes		yes	
MAP2K1/MEK1	yes			
MAP2K2/MEK2	yes			
MAPK1/ERK2	yes			
MAPK3/ERK1	yes			
MET	yes	yes	yes	
MLH1	yes	yes		
MPL	yes			
MTOR	yes	yes		
MYC	yes		yes	
NF1	yes	yes		
NFE2L2	yes			
NOTCH1	yes			
NPM1	yes			
NRAS	yes			
NTRK1	yes			yes
NTRK3	yes			

PDGFRA	yes	yes	yes	
PIK3CA	yes		yes	
PTEN	yes	yes		
PTPN11	yes			
RAF1	yes		yes	
RB1	yes	yes		
RET	yes			yes
RHEB	yes			
RHOA	yes			
RIT1	yes			
ROS1	yes			yes
SMAD4	yes	yes		
SMO	yes			
STK11	yes	yes		
TERT	yes			
TP53	yes	yes		
TSC1	yes	yes		
VHL	yes	yes		

Supplementary Table 2: Demographic characteristics within T790M subclonal and clonal groups stratified by treatment arm.

Demographic characteristic		Clonal [a] Osimertinib (N=92)	Clonal [a] Chemotherapy (N=27)	Sub-clonal [a] Osimertinib (N=39)	Sub-clonal [a] Chemotherapy (N=26)	Total (N=184)
Age (years)	n	92	27	39	26	184
	Mean	61.2	63.1	59.3	59	60.7
	SD	12.46	12.64	11.79	13.28	12.44
	Median	62	67	58	60	62
	Min	25	33	36	20	20
	Max	82	80	82	80	82
Age group (years), n (%)	<50	16 (17.4)	5 (18.5)	7 (17.9)	6 (23.1)	34 (18.5)
	>=50-<65	38 (41.3)	7 (25.9)	19 (48.7)	12 (46.2)	76 (41.3)
	>=65-<75	21 (22.8)	11 (40.7)	9 (23.1)	3 (11.5)	44 (23.9)
	>=75	17 (18.5)	4 (14.8)	4 (10.3)	5 (19.2)	30 (16.3)
Sex, n (%)	Male	34 (37.0)	7 (25.9)	14 (35.9)	7 (26.9)	62 (33.7)
	Female	58 (63.0)	20 (74.1)	25 (64.1)	19 (73.1)	122 (66.3)
Race, n (%)	White	37 (40.2)	9 (33.3)	18 (46.2)	10 (38.5)	74 (40.2)
	Black or African American	3 (3.3)	0	0	0	3 (1.6)
	Asian	51 (55.4)	18 (66.7)	19 (48.7)	16 (61.5)	104 (56.5)
	Native Hawaiian or other Pacific Islander	0	0	0	0	0
	American Indian or Alaska Native	0	0	0	0	0
	Other	1 (1.1)	0	2 (5.1)	0	3 (1.6)
Smoking status, n (%)	Never	60 (65.2)	16 (59.3)	27 (69.2)	21 (80.8)	124 (67.4)
	Current	5 (5.4)	1 (3.7)	3 (7.7)	0	9 (4.9)
	Former	27 (29.3)	10 (37.0)	9 (23.1)	5 (19.2)	51 (27.7)

[a] If a patient with the relative T790M VAF greater than 30% then the patient will be in subgroup of 'clonal' otherwise the patient will be in subgroup of 'subclonal'.

Supplementary Table 3: Comparisons for demographic characteristics between T790M subclonal and T790M clonal subgroups of AURA3 patients.

Demographic characteristic		Clonal [a] (N=119)	Sub-clonal [a] (N=65)	2-sided p-value [b]
Age group (years), n (%)	<65	66 (55.5)	44 (67.7)	0.1058
	>=65	53 (44.5)	21 (32.3)	
Sex, n (%)	Male	41 (34.5)	21 (32.3)	0.7685
	Female	78 (65.5)	44 (67.7)	
Race, n (%)	Asian	69 (58.0)	35 (53.8)	0.5884
	Non-Asian	50 (42.0)	30 (46.2)	
Smoking status, n (%)	Never	76 (63.9)	48 (73.8)	0.1675
	Current/Former	43 (36.1)	17 (26.2)	

[a] If a patient with the relative T790M VAF greater than 30% then the patient will be in subgroup of 'clonal' otherwise the patient will be in subgroup of 'subclonal'.

[b] 2-sided p-value is based on Chi-square test for the comparisons. Statistical comparison is based on the non-missing values.

Supplementary Table 4: List of pathways and genes included in the GH360 panel and known to be involved in TKI-resistance. RTK, receptor tyrosine kinase.

Pathway	Gene	Alteration
PI3K	PIK3CA	oncogenic mutations and gene amplifications
	PTEN	oncogenic mutations
RTK (bypass pathway activation)	ERBB2 amp	gene amplifications
	FGFR1 amp	gene amplifications
	MET amp	gene amplifications
MAPK	NF1	oncogenic mutations
	KRAS	oncogenic mutations
	BRAF	oncogenic mutations
	MAP2K1	oncogenic mutations
Cell cycle	CCNE1 amp	gene amplifications
	CCND2 amp	gene amplifications
	CCND1 amp	gene amplifications
	CDK6 amp	gene amplifications
	CDKN2A	oncogenic mutations
	CDK4 amp	gene amplifications

Supplementary Table 5: List of pathogenic alterations in genes known to be involved in TKI resistance presented in Figure 3C.

GENE	PROTEIN CHANGE	CDS CHANGE
BRAF	amp	amp
BRAF	N581S	c.1742A>G
CCND1	amp	amp
CCND2	amp	amp
CCNE1	amp	amp
CDK4	amp	amp
CDK6	amp	amp
CDKN2A	C72*	c.216C>A
CDKN2A	R80*	c.238C>T
CDKN2A	p.Glu27fs	c.77dupA
CDKN2A	p.Ala4_Pro11del	c.9_32delGGCGGCGGGGAGCAGCATGGAGCC
ERBB2	amp	amp
FGFR1	amp	amp
KRAS	amp	amp
MAP2K1	P124L	c.371C>T
MET	amp	amp
NF1	p.Asp424fs	c.1270_1286delGATTGGTGGCCTAAGAT
NF1	R461Q	c.1382G>A
NF1	H501Y	c.1501C>T
NF1	p.Tyr80fs	c.237dupA
NF1	N1054D	c.3160A>G
NF1	V1242G	c.3725T>G
NF1	K1283R	c.3848A>G
NF1	p.Glu2339fs	c.7014delT
NF1	D2375N	c.7123G>A
NF1	p.Lys2422fs	c.7246_7264dupCTACTAACTCTGGTTAACA
PIK3CA	amp	amp
PIK3CA	N345K	c.1035T>A
PIK3CA	E542K	c.1624G>A
PIK3CA	E545K	c.1633G>A
PIK3CA	Q546E	c.1636C>G
PIK3CA	M1043I	c.3129G>C
PIK3CA	H1047Y	c.3139C>T
PIK3CA	H1047R	c.3140A>G
PTEN	R130Q	c.389G>A
PTEN	p.Arg172fs	c.514_523delAGGCGCTATG
PTEN	Y225*	c.675T>G
PTEN	p.Asp236fs	c.705delA
PTEN	splice acceptor variant	c.802-2A>T

* amp reported if copy number above 2

Supplementary Table 6: Primers/probes details. Adapter sequences for binding indexing primers are shown in capitals in the amplicon NGS primers.

Application	Primer/probe	Primer/probe name	Sequence
amplicon NGS	primer	PIK3CA_F	TCGTCGGCAGCGTCAGATGTGTATAAGAGACAGtgatgcttggctctggaatgc
amplicon NGS	primer	PIK3CA_R	GTCTCGTGGGCTCGGAGATGTGTATAAGAGACAGtgcatgctgtttaattgtgtggaa
ddPCR	primer	PIK3CA H1047R_F	TCGAAAGACCCTAGCCTTAGA
ddPCR	primer	PIK3CA H1047R_R	TGTGTGGAAGATCCAATCCAT
ddPCR	probe	PIK3CA H1047R_WT	/5HEX/TG+CA+C+A+T+CAT+GG/3IBFQ/
ddPCR	probe	PIK3CA H1047R_mut	/5-6FAM/TG+CA+C+G+TCA+TG/3IBFQ/
ddPCR	primer	EGFR T790M_F	CATCTGCCTCACCTCCAC
ddPCR	primer	EGFR T790M_R	TCTTTGTGTTCCCGGACATAG
ddPCR	probe	EGFR T790M_WT	/5HEX/CA+TC+A+C+GC+A+GC/3IABkFQ/
ddPCR	probe	EGFR T790M_mut	/5-6FAM/TCA+TC+A+T+GC+A+GC/3IABkFQ/

Supplementary Table 7: Amplicon sequencing summary.

**Sample**	**Chromosome**	**Start**	**Stop**	**#Reads**	**#Aligned**	**%Aligned**
PC9	3	178951955	178952143	247378	246728	99.737
PC9-PIK3CA-H1047R	3	178951955	178952143	228567	227833	99.679
